# A Novel Multi-Phosphonate Surface Treatment of Titanium Dental Implants: A Study in Sheep

**DOI:** 10.3390/jfb5030135

**Published:** 2014-09-10

**Authors:** Marcella von Salis-Soglio, Stefan Stübinger, Michéle Sidler, Karina Klein, Stephen J. Ferguson, Käthi Kämpf, Katalin Zlinszky, Sabrina Buchini, Richard Curno, Péter Péchy, Bjorn-Owe Aronsson, Brigitte von Rechenberg

**Affiliations:** 1 Musculoskeletal Research Unit, Equine Hospital, Vetsuisse Faculty, University of Zurich, Winterthurerstrasse 260, Zürich 8057, Switzerland; E-Mails: msalis@ethz.ch (M.S.-S.); stefan.stuebinger@cabmm.uzh.ch (S.S.); msidler@vetclinics.uzh.ch (M.S.); kklein@vetclinics.uzh.ch (K.Kl.); kkaempf@vetclinics.uzh.ch (K.Kä.); zlin@vetpath.uzh.ch (K.Z.); 2 Center of Applied Biotechnology and Molecular Medicine (CABMM), Vetsuisse Faculty, University of Zurich, Winterthurerstrasse 190, Zürich 8057, Switzerland; 3 Veterinary Anaesthesia Services Int (VAS), Zürcherstrasse 39, Winterthur 8400, Switzerland; 4 Institute for Biomechanics, ETH Zürich, Vladimir-Prelog-Weg 1-5/10, Zürich 8093, Switzerland; E-Mail: sferguson@ethz.ch; 5 Nano Bridging Molecules SA, Rte Cité Ouest 2, Gland 1196, Switzerland; E-Mails: sabrina.buchini@nbmolecules.com (S.B.); richard.curno@nbmolecules.com (R.C.); peter.pechy@nbmolecules.com (P.P.); bjorn-owe.aronsson@nbmolecules.com (B.-O.A.)

**Keywords:** surface treatment, multi-phosphonate, bone formation, removal torque test, bone-to-implant-contact, scanning electron microscopy, osseointegration

## Abstract

The aim of the present study was to evaluate a new multi-phosphonate surface treatment (SurfLink^®^) in an unloaded sheep model. Treated implants were compared to control implants in terms of bone to implant contact (BIC), bone formation, and biomechanical stability. The study used two types of implants (rough or machined surface finish) each with either the multi-phosphonate Wet or Dry treatment or no treatment (control) for a total of six groups. Animals were sacrificed after 2, 8, and 52 weeks. No adverse events were observed at any time point. At two weeks, removal torque showed significantly higher values for the multi-phosphonate treated rough surface (+32% and +29%, Dry and Wet, respectively) compared to rough control. At 52 weeks, a significantly higher removal torque was observed for the multi-phosphonate treated machined surfaces (+37% and 23%, Dry and Wet, respectively). The multi-phosphonate treated groups showed a positive tendency for higher BIC with time and increased new-old bone ratio at eight weeks. SEM images revealed greater amounts of organic materials on the multi-phosphonate treated compared to control implants, with the bone fracture (from the torque test) appearing within the bone rather than at the bone to implant interface as it occurred for control implants.

## 1. Introduction

Osseointegrated dental implants were internationally accepted after the Toronto conference held in 1982, mainly based on the early and pioneering investigations of Brånemark *et al*. in Sweden and Schroeder *et al*. in Switzerland [1]. In the following decades, the successful integration of titanium dental implants in bone has been extensively documented and supported by innumerable *in vitro* and *in vivo* studies, as well as long-term clinical experience [2,3,4]. Titanium is currently regarded as the implant material of choice in respect to strength, biocompatibility, and stability against corrosion or degradation *in vivo* [5]. However, early and late stage bone formation and remodeling around titanium dental implants is not well understood. Complications and implant failures due, to e.g., aseptic loosening is a prevailing problem. Different technological approaches to improve biocompatibility are reported in the literature, to date none has proven itself to be the solution [6].

More than 30 years after the first osseointegration conference in 1982, close to 600 different implant designs by at least 146 different manufacturers [7] offer innovative macro-, micro-, and nano-structured implant designs with numerous surface modifications and coatings to improve osseointegration and guarantee long-term success of the bone anchored implants [8,9,10]. Each of these implant systems are designed to fulfill individual clinical indications spanning single tooth reconstructions to partially, or fully edentulous jaws [11,12]. Some of these implant systems have already shown clinical success rates of up to 99% [13,14]. Despite these auspicious results in primarily healthy patients, the steady rise of patients with compromised bone conditions due to, e.g., metabolic disorders, smoking, obesity [15], or irradiation therapies demand continuing refinements in implant designs, surface characteristics, surgical procedures, and loading protocols [16,17]. Additionally increasing demand for reduced time protocols and for higher aesthetical standards, as well as consolidated findings in the biological understanding of periimplant hard and soft tissue processes require innovative implant materials and surface treatment regimens [18,19]. Such innovative surface treatments are expected to improve osseointegration and reduce the risk of long term failure.

Various review articles covering both *in vitro* and *in vivo* studies have shown that a moderate surface roughness provides favorable conditions for enhanced bone integration with increased peri-implant osteoconduction and osteogenesis [20,21,22].

It has been shown that phosphorous containing nanostructured or biomimetic surface coatings, as well as so-called bioactive implants, may offer a direct chemical bonding between bone and implant with improved osseointegration [23,24,25,26,27,28]. However, polyphosphoric acid and polyphosphates are easily hydrolysed in the body and, thus, quickly removed from the implant surface.

Phosphonates are analogues of phosphates whereby one oxygen atom has been replaced by a carbon atom rendering the molecule stable towards enzymatic hydrolysis [29]. Phosphonates are highly polar molecules and, as such, extremely hygroscopic and, thus, will provide improved surface wettability. Increased wettability is thought to accelerate healing and early osseointegration [30].

*In vitro* and small animal studies have shown evidence that peptides (e.g., RGD and Bone Morphogenic Proteins) functionalized with monophosphonates can be effectively anchored onto titanium surfaces [31,32,33]. Enhanced cell adhesion and bone fixation prompted by these peptides were demonstrated.

The aim of the present study was to investigate the biological influence of a novel surface treatment, made up of multi-phosphonated molecules (SurfLink^®^), on the osseointegration process of commercially rough and machined titanium implant surfaces by biomechanical and histological evaluations after 2, 8, and 52 weeks integration in sheep, as well as by Scanning Electron Microscopy (SEM) analysis of retrieved implants after 52 weeks.

## 2. Results and Discussion

### 2.1. Results

#### 2.1.1. Implants and Surface Characteristics

Multi-phosphonate treated and control implants were successfully prepared. A selection of implants was surface characterized by X-ray Photo-electron Spectroscopy (XPS) and the results were in agreement with previously published data [34].

#### 2.1.2. Surgery and Postoperative Period

Surgery and anesthesia were uneventful. No signs of lameness or other discomfort were seen in animals. No infections occurred. All implants could be placed without any major complications and with primary stability. Because of icteric tissue, one sheep had to be removed from the study during surgery and was replaced prior to implantation.

#### 2.1.3. Macroscopical and Radiological Evaluation

Two adjacent machined implants (1 × multi-phosphonated surface Dry 52 weeks; 1 × control 52 weeks) in the same animal were found next to the implantation site in the muscle. These implants were judged to have been misplaced (placed using a broken drill guide) and, thus, were excluded from further analysis. All other implants were clinically stable. Macroscopically, a periosteum-like soft tissue layer was visible over the cover caps after eight weeks. After 52 weeks several cover caps, predominantly in positions 1–4 (most caudally), were overgrown with bony tissue. Radiographs in two directions demonstrated that all implants except two were correctly placed (*n =* 430). No signs of peri-implant lysis, inflammation or fractures were observed.

#### 2.1.4. Microradiographic Evaluation

A small seam of non-calcified tissue was seen at all implant interfaces after two weeks. After eight and 52 weeks a radio-dense zone adjacent to the implant, showed no sign of bone resorption or fibrous encapsulation. As this observation was in agreement with the images presented from the ground sections stained with toluidine blue, additional evaluation of microradiographs was renounced (images are not shown).

#### 2.1.5. Torque-Test Evaluation

The groups with the rough surfaces showed higher torque values compared to the machined groups. While removal torque values were highest at 52 weeks for all groups, notable differences between groups at two and eight weeks were found only for the rough surface implants (Table 1).

**Table 1:** Average biomechanical data: removal torque (N·mm) and rotational stiffness (N·mm/°). SE = standard error.

Group		Removal Torque (N·mm)		Stiffness (N·mm/°)	
		Average	SE	Average	SE
RD	2 weeks	452.3	29.0	155.5	9.7
	8 weeks	1055.6	39.2	180.9	4.5
	52 weeks	1286.9	71.0	155.5	12.5
RW	2 weeks	516.5	46.1	155.9	9.1
	8 weeks	920.4	73.2	186.7	10.1
	52 weeks	1246.4	103.6	156.3	17.5
RC	2 weeks	450.8	37.8	140.0	9.1
	8 weeks	1035.0	45.4	194.2	6.9
	52 weeks	1267.5	45.1	138.9	12.7
MD	2 weeks	251.5	35.0	125.6	17.9
	8 weeks	283.3	23.6	105.3	11.0
	52 weeks	692.5	53.6	86.9	9.5
MW	2 weeks	277.4	31.9	117.4	13.7
	8 weeks	290.5	29.3	109.1	8.7
	52 weeks	717.5	80.4	96.6	11.5
MC	2 weeks	274.4	22.4	123.5	14.3
	8 weeks	267.7	20.5	119.9	15.5
	52 weeks	758.2	107.5	104.8	16.1

**Figure 1:**
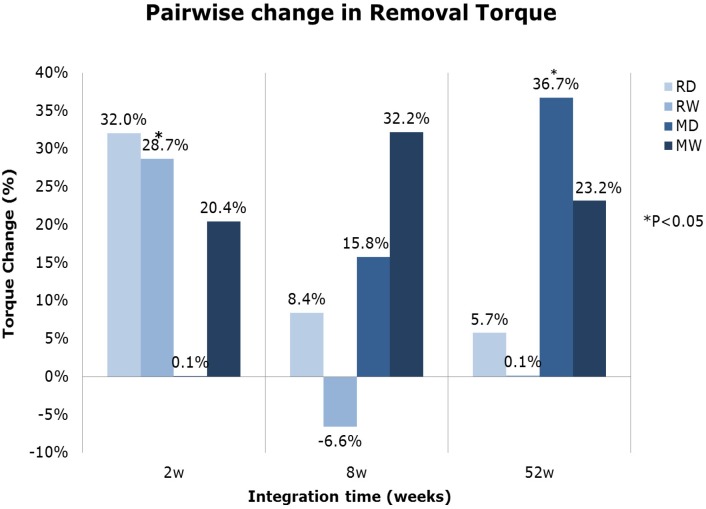
Mean pairwise relative difference in removal torque values of multi-phosphonate treated *versus* control implants by time. Statistically significant differences are indicated by an asterisk (* *p <* 0.05, one-sample two-sided Student *t*-test).

A further examination of the pair-wise histograms for removal torque values (Figure 1) showed a general increase of removal torque values for the multi-phosphonate treated implants, compared to control implants. Removal torque was significantly higher for the multi-phosphonate treated rough Wet (RW) implants at two weeks (*p =* 0.036, from pair-wise comparison) and for the multi-phosphonate treated machined Dry implants (MD) at 52 weeks (*p =* 0.025, from pair-wise comparison).

**Figure 2:**
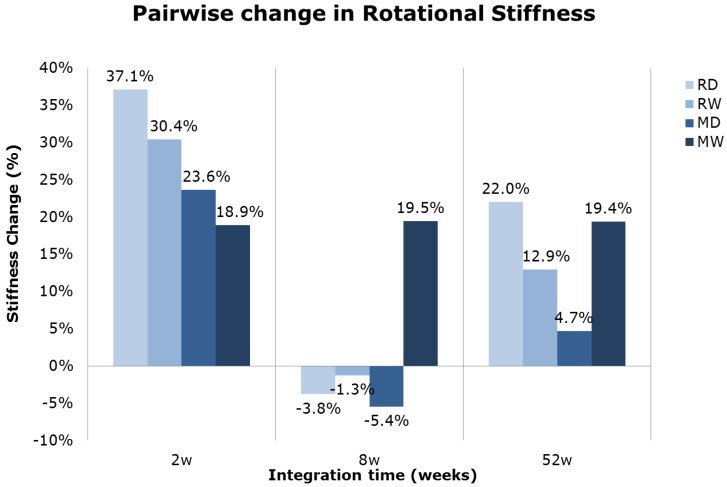
Mean pairwise relative difference in rotational stiffness values of multi-phosphonate treated *versus* control implants by time.

Rotational stiffness was also higher in groups with rough metal surfaces (Table 1). These surfaces values were highest at eight weeks and almost equal at 2 and 52 weeks. In contrast, for the machined surface groups the highest values were found at two weeks, followed by those at eight and last at 52 weeks. The pairwise analysis showed an increase in rotational stiffness for the multi-phosphonate treated implants compared to control implants, particularly at two weeks (Figure 2). The difference between the multi-phosphonate treated dry (RD) and control implants (RC) at two weeks was quasi-significant (*p =* 0.055, from pairwise comparison). Interestingly the multi-phosphonate treated machined Wet (MW) surfaces had higher removal torque and rotational stiffness at all time points (Figure 1 and Figure 2).

#### 2.1.6. Histological Evaluation and BIC

**Figure 3:**
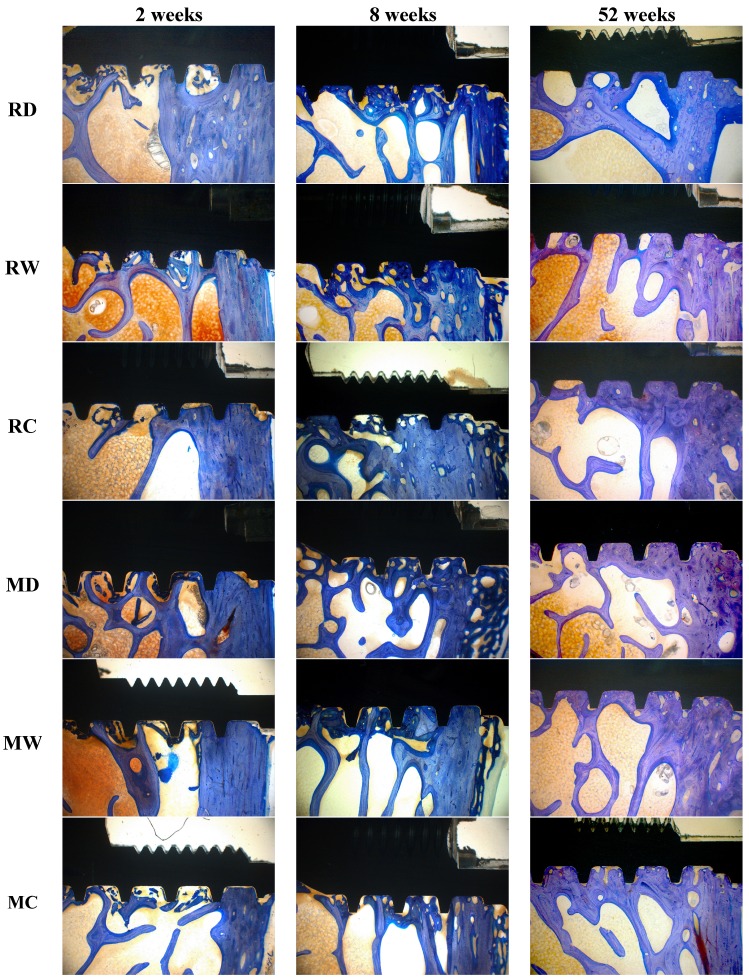
Histology images of toluidine blue stained ground sections: comparison of different groups after 2, 8, and 52 weeks of healing (original magnification 34×).

Implants of all six groups had a direct contact with the host bone after 2, 8, and 52 weeks, as evidenced by the light microscope images (Figure 3). Except for the two lost machined implants, which were surrounded by a pronounced capsule of fibrous tissue outside the bone, all other implants were partially or fully surrounded by a layer of cortical bone with trabeculae inserting into it perpendicularly.

In general, after 2 weeks, in all groups, no signs of peri-implant bone apposition or resorption was observed, a close contact between cortical bone structures and implants was seen. Especially in the apical part of implants, remnants of bone debris after implant site preparation were obvious. After 8 weeks an active zone of bone remodeling was still observed between implant threads and adjacent compartments appearing mostly as randomly oriented woven bone matrix. The gaps between primary drill channels and implant surfaces started to be bridged by bone anchors from the surrounding cancellous bone structures to the implant. After 52 weeks, reinforcement of pre-existing bone combined with physiologic bone remodeling, resulted in mainly mature lamellar bone structures around dental implants of all groups.

Statistical analysis of the average BIC measurements revealed relatively high standard errors (SE) with only significant differences (*p <* 0.05) between rough and machined implants, irrespective of surface treatment, after 2, 8, and 52 weeks.

The Total BIC was lower at eight weeks than at two or 52 weeks on all surfaces suggesting that an active bone remodeling was still taking place (Table 2).

Cortical average BIC values substantially declined over time on machined surfaces, while on rough surfaces cortical BIC values tended to stabilize or increase slightly. High cortical BIC (above 60%) was present at two weeks on all surfaces (Table 2). The multi-phosphonate Wet treated surfaces in particular (RW and MW) showed the highest, although not significant, BIC in the cortical bone at this early time point.

In cancellous bone, average BIC values on rough surfaces were initially quite high (above 70%), changing very little with time (Table 2). On all machined surfaces a substantial increase of the cancellous BIC was observed at 52 weeks.

**Table 2:** Average bone to implant contact (BIC) (%). SE = standard error.

Group		Cortical		Cancellous		Total	
		Mean	SE	Mean	SE	Mean	SE
RD	2 weeks	72.3	8.1	84.9	3.3	84.4	2.9
	8 weeks	61.9	6.1	72.1	4.5	66.0	3.6
	52 weeks	84.0	4.5	73.9	7.0	76.4	4.4
RW	2 weeks	78.8	5.2	76.3	5.4	73.5	6.1
	8 weeks	63.5	4.6	76.8	4.0	71.5	5.4
	52 weeks	72.3	7.2	80.6	5.3	77.5	3.4
RC	2 weeks	75.9	6.4	81.3	3.0	81.4	2.1
	8 weeks	65.2	5.9	71.9	4.5	68.8	4.7
	52 weeks	80.7	4.3	76.1	5.3	76.0	3.9
MD	2 weeks	64.2	7.2	26.0	3.7	33.9	3.9
	8 weeks	44.0	5.7	27.9	4.5	32.1	3.6
	52 weeks	44.5	5.3	52.2	4.8	46.6	4.7
MW	2 weeks	75.5	6.5	36.6	3.4	45.2	4.3
	8 weeks	47.3	9.0	33.4	4.8	37.1	6.6
	52 weeks	45.5	7.6	50.8	6.5	47.1	6.0
MC	2 weeks	67.3	8.2	36.0	4.8	38.0	4.3
	8 weeks	51.7	6.2	27.2	4.0	34.4	4.1
	52 weeks	42.3	3.7	53.6	5.4	48.6	5.2

**Figure 4:**
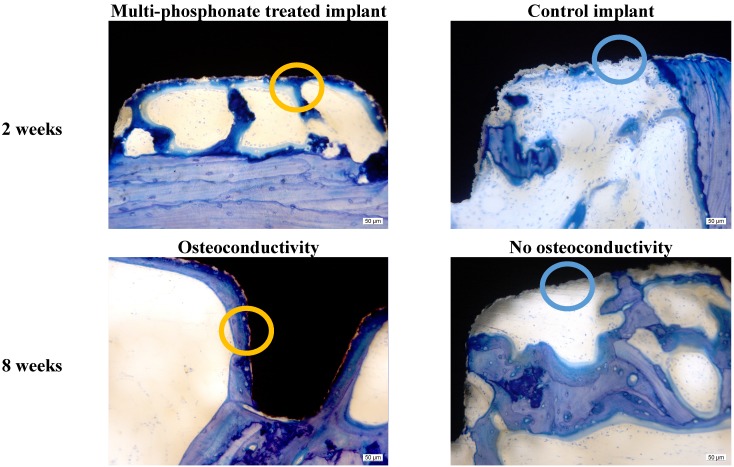
High magnification of toluidine blue stained histology ground sections of osteoconductive multi-phosphonate treated implants and non osteoconductive control implants after two- and eight-week healing in sheep.

**Figure 5:**
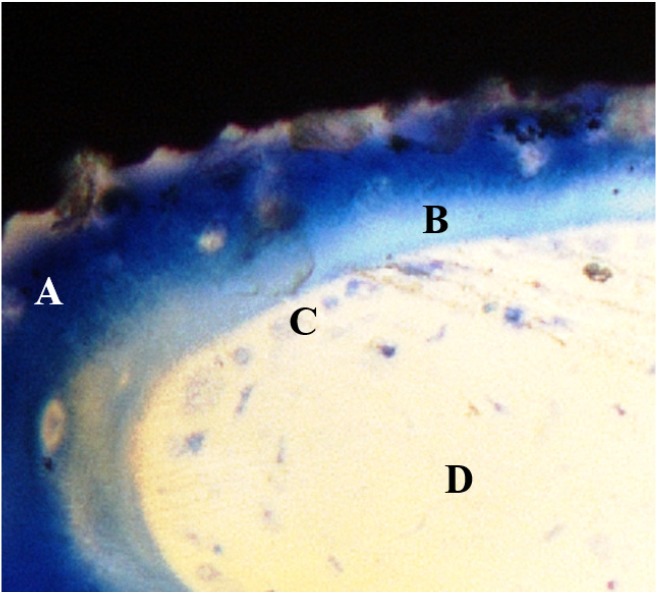
Process of bone formation on a multi-phosphonate treated implant at 2 weeks healing in sheep. A: dark blue, mineralised bone; B: light blue, matrix osteoid; C: light blue dots, osteoblasts; D: white, bone marrow.

Microscope images at higher magnification of selected multi-phosphonate treated rough Dry (RD) and control (RC) implants showed that contact points originating from the surrounding trabecular bone are formed on both multi-phosphonate treated and control implants at 2 and 8 weeks (Figure 4). Bone cells spread out from these contact points on the multi-phosphonate treated implants but not on the control implants. A close-up of the multi-phosphonate treated implant shows mineralised bone, osteoid and lining osteoblasts directly at the surface (Figure 5) as early as two weeks (Figure 4). This behavior was not observed on control implants at either two or eight weeks (Figure 4).

#### 2.1.7. Histomorphometric Evaluation

**Figure 6:**
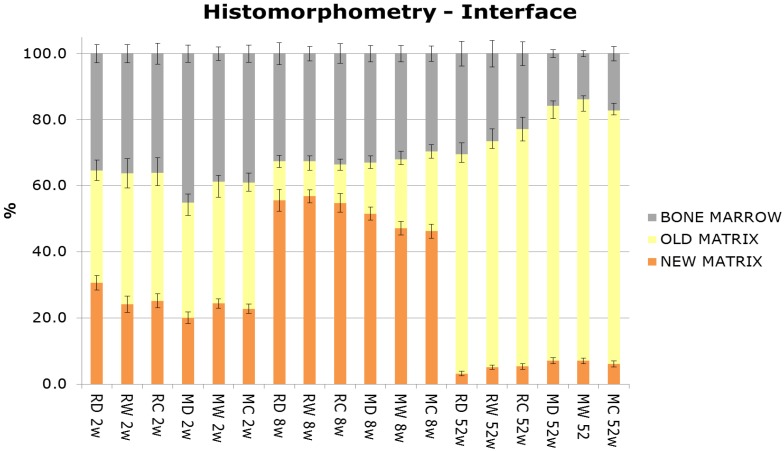
Histomorphometrical analysis (%) of the interface compartment adjacent to the implant surface. Mean values by group and time.

**Figure 7:**
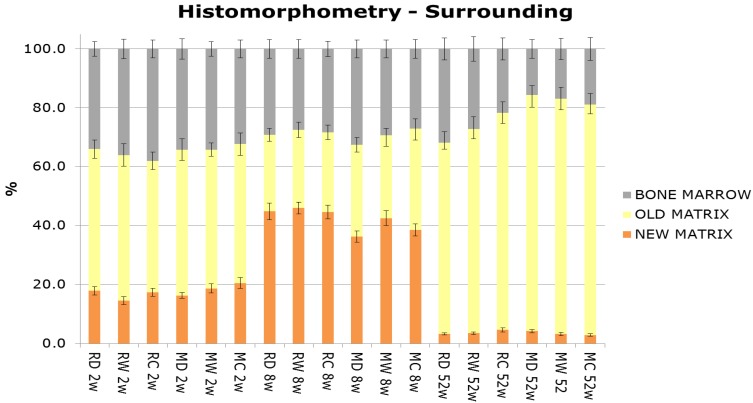
Histomorphometrical analysis (%) of the surrounding compartment adjacent to the implant surface. Mean values by group and time.

New bone matrix was observed at the implant interface, as well as at the surrounding bone compartments, in all groups and all time points (Figure 6 and Figure 7). After 2, 8, and 52 weeks, increased new bone formation was seen at the interface of rough and machined implants relative to surrounding bone. Overall there was a marked increase in new bone formation at the interface and the surrounding bone from 2 to 8 weeks, followed by a decrease at 52 weeks, which corresponds to normal physiological bone remodeling. Furthermore, independent of surface treatment and time, more old bone matrix (lamellar) was seen at the interface and surrounding compartments of the machined surfaces in comparison to the rough surfaces.

**Figure 8:**
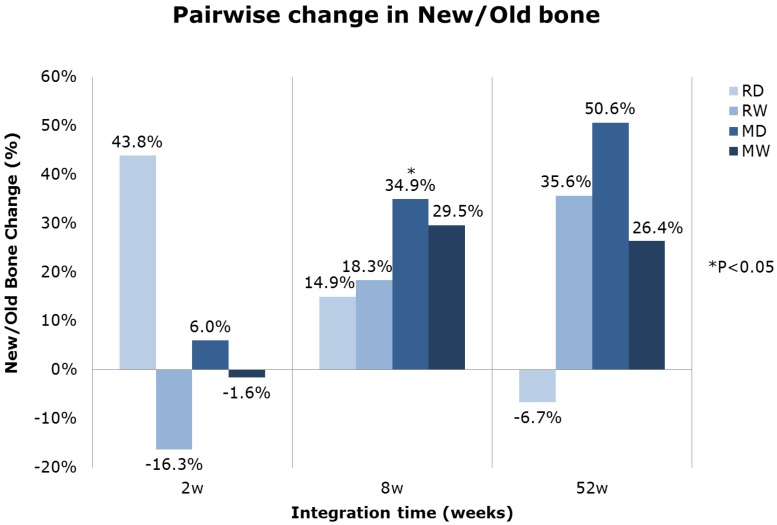
Mean pairwise relative difference in new-to-old bone changes of multi-phosphonate treated *versus* control implants by time.

A pair-wise statistical analysis of the overall histomorphometric data revealed a clear positive trend for an increase in new-old bone ratio for all multi-phosphonate treated surfaces at 8 weeks (Figure 8). In particular, the multi-phosphonate treated Dry machined group (MD) presented a statistically significant increase (*p =* 0.039).

#### 2.1.8. Scanning Electron Microscopy (SEM) Evaluation of Torque Tested Implants

**Figure 9:**
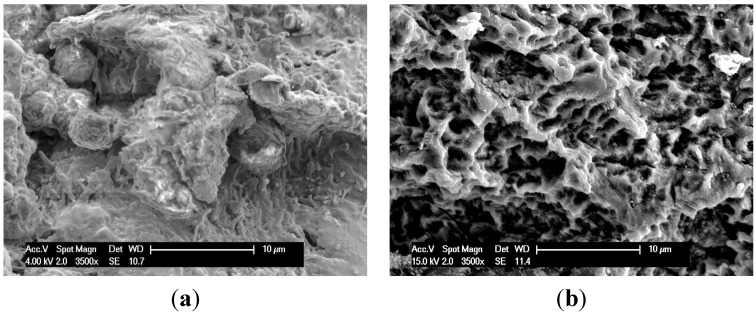
SEM of (**a**) multi-phosphonate rough Dry (RD) and (**b**) control (rough) implants retrieved after 52 weeks (3500×). On RD, remnants of adhering trabecular bone is observed, while on control implant only the original rough implant surface is observed.

Figure 9 shows the SEM images of multi-phosphonate treated rough Dry (RD) and control implants at different magnifications after 52 weeks. Similar features were seen on all implant surfaces but in greater abundance on the multi-phosphonate treated implants. Bone remodeling was observed at several places on the implant both in the cortical and in the cancellous bone regions as evidenced by the presence of mineralized filaments as well as remnants of lamellar bone.

### 2.2. Discussion

The aim of this animal study in sheep was to investigate the safety and efficacy of a novel multi-phosphonate surface treatment, SurfLink^®^, on commercially rough and machined titanium dental implants after 2, 8, and 52 weeks. While the healing period of two to eight weeks should correspond to the phase of early implant integration, results after 52 weeks should provide information for the mid- to long-term outcome [35].

This novel surface treatment was shown to covalently bind to the oxide layer of the titanium implant surface [34,36]. In virtue of its chemical structure the treatment is stable against chemical and enzymatic hydrolysis, and is, thus, permanently attached to the implant surface under physiological conditions. Whereas conventional micro-structured and moderately rough dental implants rely on mechanical interlocking [37], this surface treatment was designed to provide a linker which results in a rapidly established and stable chemical bone-to-implant interface. In a preliminary study in a rat model, significant increases in early fixation were observed [38]. The first clinical results with multi-phosphonate treated dental implants showed at one-year post-loading an excellent outcome with no implant failures and maintaining marginal bone levels, while control implants lost a significant amount of marginal bone after loading [39].

The experimental setup for this study was a pelvic sheep model, as used in previous studies investigating the osseointegration properties of various implant designs and surfaces [40]. Sheep animal models are well-established in orthopaedic research for analyzing fracture healing, new osteosynthesis techniques and also osseointegration of implants, because they exhibit a very similar lamellar bone structure to humans [41,42,43]. Furthermore, bone from the sheep pelvic region resembles a bone structure similar to the human mandible mimicking a more cancellous structure in the cranial part with increasing cortical thickness towards the caudal part. Additionally, overlying muscle tissues also reflect some slight intermittent biomechanical forces on the supracrestal implant parts. This sheep model was found to be economical in its use of animals, it provided the intra- and inter individual data for statistical comparison and it was associated with a zero failure rate of surgery [44]. In contrast to an alternative and also well-established animal model using beagle dogs, the sheep model used in this study avoided an ongoing and stimulating bony process by previous manipulation through dental extractions. In addition it excludes the 2.5-fold higher bone remodeling rate and post-operative complications like poor mouth hygiene commonly associated with other animal models [45]. However, the soft tissue interface and microbiological issues could not be scrutinized in this model. Together with the fact that the pelvic shaft model presents a prosthodontically unloaded experimental set-up in periphery bone, this can be seen as critical drawback for evaluating long-term success in a dental clinical environment [46]. Nevertheless, since animal models are always an approximation, it is wise to take a stepwise approach and first test biocompatibility and osseointegration under non-complicated conditions before adding the next complex question of soft tissue and bacterial interference.

Due to its phosphonic functional groups, the multi-phosphonate surface treatment has been found to increase hydrophilicity and wettability of implant surfaces and thus an increase in affinity of blood, proteins and bone cells for the implant surface is expected. Once on the implant surface, bone cells quickly spread along the multi-phosphonate treated implant as shown by Figure 4 (2 weeks). The increased bone in contact with the multi-phosphonate treated implant results in greater biomechanical implant fixation.

Biomechanical stability of implants with the multi-phosphonate treatment tended to result in superior torque values after 2 weeks, especially when combined with a rough implant surface, which resulted in significantly (*p* = 0.036, RW, pairwise comparison) higher removal torque values (Figure 1). Additionally, the multi-phosphonate treated Wet machined implants (MW) showed tendencies for greater torque and stiffness values at all time points when compared to control machined implants (MC) (Figure 1 and Figure 2). These results corroborate previously published data from a study in rats [38], whereby a significant increase in pull-out strength (+38%, *p <* 0.01) for the multi-phosphonate treated cylinders was observed.

Biomechanical data was confirmed by the histology results, which showed positive tendencies for the multi-phosphonate treated implants.

While cortical bone generally provides sufficient primary stability, increased implant failure rates are often observed in areas with loose trabecular bone like in the posterior maxilla [47]. Hence, evaluation of the BIC was also conducted considering cortical and cancellous bone structures separately. After 8 weeks ongoing active peri-implant bone remodeling processes led to an overall decline of Total BIC (Table 2) in all groups indicating the interval of reduced implant stability between the second and third healing phase during the osseointegration process. Nevertheless, the results showed that at 8 and 52 weeks all the multi-phosphonate treated surfaces had tendencies for increased Total BIC (from pair-wise analysis, data not shown).

Histomorphometrical evaluation exhibited significant production of new bone at the interface of all implant surfaces between two and eight weeks (Figure 6), also indicative of normal active bone remodeling taking place after implantation. An increased new-old bone ratio was observed on the multi-phosphonate treated rough Dry implants (RD: +44%, pair-wise analysis) at 2 weeks and on the multi-phosphonate treated machined Dry surfaces at eight weeks (MD: +35%, *p =* 0.039, pair-wise analysis, Figure 8). Indeed the higher magnification histological images showed that the multi-phosphonate treated surfaces are osteoconductive, with bone cells quickly spreading out from contact points to cover the implant surface, resulting in greater woven bone matrix formation on the implant. A close-up of the multi-phosphonate treated implant shows mineralised bone, osteoid and lining osteoblasts directly at the surface (Figure 5) as early as two weeks (Figure 4). This behavior was not observed on control implants at either two or eight weeks (Figure 4).

The positive tendencies seen in the biomechanical and BIC analyses of the multi-phosphonated treated surfaces were supported by the SEM images of the bone to implant interface (Figure 9). On the multi-phosphonated treated implants bone remodeling and mineral deposition were observed at several places, both in cortical and cancellous bone. Furthermore, at 52 weeks, rupture after torque testing occurred within the mature lamellar bone rather than at the bone to implant interface, suggesting a strong bond of the surrounding bone to the multi-phosphonated treated implant, as also seen in Figure 5. On control implant surfaces the same kind of organic features were observed, but less abundantly than on the multi-phosphonated treated implants. Inspection of the lamellar bone remnants showed fracture at the bone to implant interface more frequently than within bone.

To the authors knowledge there are no commercially available similar technologies to the multi-phosphonate surface treatment. Hydroxyapatite (HA) coating is seen as the gold standard of biomimetic implant surface coatings. However, while preclinical testing has been mostly positive, some studies have reported less favorable results [8,48]. To date, clinical trials have failed to show a difference in clinical outcome for HA treated dental implants. Furthermore, long term HA-coated implant stability is uncertain [49] and it remains clinically unproven [50].

The first clinical results of multi-phosphonate treated implants were recently reported in the literature [39]. One year post-loading results from a Randomized Controlled clinical Trial (RCT) showed that the multi-phosphonate treated implants were well osseointegrated. Furthermore, the data suggested a tendency for reduced peri-implant bone loss. The difference between multi-phosphonate treated and control implants was quasi-significant (*p =* 0.057). It must be noted that the number of patients included in the analysis was only 21. Long-term follow-ups will be reported at three years and five years post-loading.

Statistical analysis of the biomechanical and BIC data in this animal study showed significant differences (*p <* 0.05 and *p <* 0.01, respectively, from ANOVA statistical analysis) between rough and machined implants, irrespective of the treatment after 2, 8, and 52 weeks. This is in accordance with the well-known concept that surface micro-roughness of implants in the range of 1–10 µm affects the rate of osseointegration and mechanical fixation by maximal interlocking of bone matrix and implant surface [14,51].

It has been noted that there is a lack of statistically significant differences in BIC between treated and control groups for either surfaces. It must be considered that this is an optimized experimental design in an unloaded and uncomplicated situation and the commercial titanium implant system used for all groups enjoys a high rate of clinical success [52]. From our experience with the same animal model a drop in BIC and torque values of up to 50% can be seen depending on implant design and material of dental implants [7]. In clinical reality, implant failures are dependent on many factors, and not just the implant itself. Soft tissue integrity, bacterial contamination and the surgeon’s manual capabilities are among these factors that may finally determine if dental implants belong to the category of 2%–5% failures or success [53]. Indeed, the clinical results obtained with the multi-phosphonate treated implants [39] suggest that this novel treatment may affect the early integration and immediate sealing of an implant, particularly in a compromised bone situation (e.g., soft tissue-bone, bone augmentation), thus, minimizing the overall and individual implant failure rates.

## 3. Experimental Section

### 3.1. Implants and Surface Characteristics

Altogether 432 screw type, self-cutting Titanium (Ti) grade 4 implants (SPI^®^Element, Thommen Medical AG, Waldenburg, Switzerland) with a diameter of 3.5 mm and a length of 8 mm, were manually inserted by two experienced surgeons. Implants had either a moderately rough (*n =* 216) surface (sandblasted and acid-etched, *R*_a_: 2.193 ± 0.46 µm) or a machined (*n =* 216) surface (*R*_a_ typically around 0.3–0.4 µm [54]). Initially all implants were cleaned according to the SurfLink^®^ pre-treatment protocol (Nano Bridging Molecules SA, Gland, Switzerland). One third were then dried and packaged and served as control (RC and MC, Table 3), while two thirds of the implants were surface treated with multi-phosphonated molecules (SurfLink^®^), resulting in either rough or machined surfaces and stored in either dry conditions (RD and MD, Table 3) or wet conditions (RW and MW, Table 3).

**Table 3:** Group distribution.

Group ID	Surface	Treatment	Storage
RD	Rough	Multi-phosphonate	Dry
RW		Multi-phosphonate	Wet
RC		Control	Dry
MD	Machined	Multi-phosphonate	Dry
MW		Multi-phosphonate	Wet
MC		Control	Dry

All implants were packaged in double packaging and sterilized by gamma irradiation. Two implants from each group were analyzed by X-ray Photo-electron Spectroscopy (XPS) for verification of the chemical surface composition. XPS analyses were performed on an Axis Ultra spectrometer from Kratos (Kratos, Manchester, UK) equipped with a concentric hemispherical analyzer and using a monochromatized aluminium anode X-ray source (Al KR1,2 1486.6 eV, full width at half maximum, fwhm, 0.85 eV, 15 kV, 150 W). The samples were investigated under ultrahigh vacuum conditions: 10^−8^–10^−7^ Pa. Spectra were taken at a 90° take-off angle with respect to the surface. A sample area of 300 × 700 µm^2^ was analyzed with a pass energy of 80.0 eV for survey scans and 40.0 eV for high energy resolution elemental scans. The spectrometer was calibrated by using Cu2p3/2 (932.7 eV) and Au4f7/2 (84.0 eV) signals. Spectra were referenced in the C1s spectrum to C–H/C–C at 285.0 eV. Spectra were decomposed by assuming a Gaussian/Lorentzian (70/30) peak shape [55].

### 3.2. Animals and Surgical Technique

Twenty-four mature female sheep between 2 and 5 years of age, with an average weight of 79 kg (68–100 kg), were randomly allocated to the 6 groups (Table 3). Sheep were kept in small groups with free access to food and water. Regular control of well-being, pain and general condition was performed following the general guidelines for care and use of animals in research (Tierschutzverordnung TSchV SR 455/Tierschutzgesetz TSchG SR 455). The study protocol was approved by the local veterinary authority (Gesundheitsdirektion, Canton Zürich, approval No. 62/2008).

**Figure 10:**
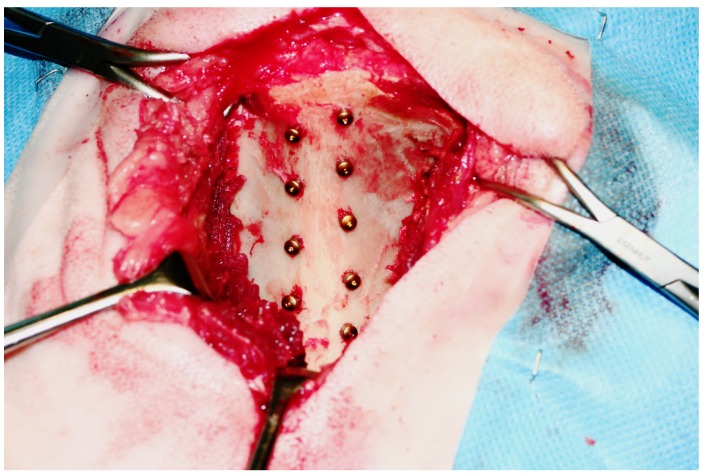
Implant positions in the pelvic bone of sheep (cranial up and caudal down).

An earlier published pelvic animal model was used for the experiment [40,41]. Briefly, all dental implants were placed in the cranial part of the left (*n =* 9) and right (*n =* 9) pelvis of each animal, alternating on either side of the linea glutea of the iliac wing. Implants were distributed in a previously randomized scheme on 9 positions of the pelvic bone, considering the more cancellous bone quality in the cranial part with increasing cortical thickness (3 mm) towards the caudal part (Figure 10). Animals were sacrificed at 2, 8, and 52 weeks after surgery. The study design aimed to achieve a statistical sample size of 12 implants per group.

### 3.3. Anaesthesia

Sedation was initiated with 0.1mg/kg Xylazin i.m. (Rompun^®^ 2%, Provet AG, Lyssach, Switzerland) and 0.01 mg/kg Buprenorphin i.m. (Temgesic^®^, Essex Chemie AG. Luzern, Switzerland). Shortly before surgery a jugular catheter was placed. 4 mg/kg Carprofen i.v. (Rimadyl^®^; Pfizer, Vertrieb Dr. Gräub AG, Bern, Switzerland) was administered for improved analgesia. Peri- and postoperative antibiosis was achieved with 30,000 I.U./kg Benzylpenicillin i.v. (Procain-Penicillin Streuli ad us. vet. G. Streuli&Co AG; Uznach, Switzerland) and 6 mg/kg Gentamicin i.v. (Vetagent^®^ ad us. vet., Veterinaria AG, Zürich, Switzerland). For prophylaxis, 3000 I.E/animal of tetanus serum (Intervet ad us. vet. Vertrieb Veterinaria AG, Zürich, Switzerland) was applied by subcutaneous single injection. Anaesthesia was then induced with 2 mg/kg ketamin i.v. (Narketan 10^®^, Vetoquinol AG, Belp-Bern, Switzerland), 0.1 mg/kg diazepam i.v. (Valium^®^, Roche Pharma AG, Rheinach, Switzerland) and 2–4 mg/kg propofol i.v. (Propofol^®^ 1% Fresenius, Fresenius Kabi AG, Stans, Switzerland). Animals were intubated in sternal recumbency and anaesthesia was maintained via inhalation with 1%–1.5% Isofloran in 100% oxygen through an appropriately sized semi-closed breathing system. Propofol was administered at a rate of 0.1–0.4 mg/kg body weight throughout the duration of the procedure as constant rate fusion. An epidural anaesthesia with 0.1 mg/kg morphinhydrochlorid (Morphin-HCL Sintetica) was administered into the epidural space to provide adequate analgesia throughout the surgery.

### 3.4. Surgical Procedure

Sheep were repositioned in a tilted sternal recumbency with laterally positioned pelvis. A standard lateral approach to the dorsal aspect of the iliac crest was performed with a skin incision in the lower third of the iliac wing and in longitudinal direction of the ileum, extending *ca.* 3 cm cranially and 10 cm caudally to the middle of the crest. The pelvic fascia was cut through and the middle gluteal muscle was detached in the ventral third of the muscle insertion, such that the tendinous insertions of the deep and middle gluteal muscles were separated from the iliac crest. Soft tissue structures were bluntly removed from the filamentous attachments of the pelvic bone and retracted caudally using Langenbeck retractors. A prefabricated template with 9 alternating holes was used to drill 5 holes ventrally and 4 holes dorsally of the linea glutea. Implant site preparation was performed according to the implant manufacturer’s recommendations and drilling protocol using standard rotating pilot and twist drills (SPI^®^VECTROdrill, Thommen Medical AG, Waldenburg, Switzerland) in ascending order (diameter). Implants were manually inserted according to the implantation scheme with a torque wrench (max. 45 Ncm). Both surgeons were blinded from implant allocation and surface treatment. Healing caps were placed and implant setting was documented with digital photographs (Figure 10).

Finally the gluteal muscles were repositioned and their tendinous insertions refixed to their origins. Fascia and subcutis were closed using a synthetic resorbable suture (Polyglactin; Vicryl 2-0; Johnson&Johnson Int., Brussels, Belgium) and the skin was closed with staples. The animal was turned over to the contralateral side and operated in an identical manner.

Postoperative treatment consisted of 0.01 mg/kg Buprenorphin i.m., TID, for the first 24 h and 30,000 I.U./kg Benzylpenicillin i.v, BID, 6 mg/kg Gentamicin i.v. SID. and 4 mg/kg Carprofen i.v. SID. for the 4 following days.

### 3.5. Animal Sacrifice and Preparation of Bone Samples for Torque-Test and Histology

Animals were sacrificed at 2, 8, and 52 weeks after surgery. Pelvic bones were harvested and a complete removal of all soft tissue structures was performed to gain free access to all implants. Specimens were inspected macroscopically and bony overgrowth was documented by digital photographs. Additionally radiographs (dorso-ventral and latero-lateral) were taken using a Faxitron machine (Faxitron X-ray System, Hewlett&Packard, Kodak X-OMAT MA Film, Kodak, France) to assess implant positions and the absence of fractures and bony lysis around the implants. Bone specimens were cut using a band saw (Stryker^®^Instruments, Kalamazoo, MI, USA) into cubes of 1.5 cm × 1.5 cm at full thickness of the pelvis including one implant each.

Implants for torque removal tests were folded in moist gauze, cooled on ice and brought to the testing lab within 24–36 h.

For the histological preparation, samples were washed in saline solution, dehydrated in a series of alcohol concentrations, defatted in xylene under vacuum, infiltrated in methylmethacrylate (methacrylacid-methylester; dibuthylphtalate and perkadox in a proportion 89.5:10:0.5) and then finally embedded in the same solution using special Teflon molds placed in a standard water bath at 30 °C. Cubes were cut in parallel to the axis of the implant using a specially designed device to ensure the proper alignment. Two ground sections, 400 µm thick for histology, were cut at the maximum diameter of the implant using a low speed diamond saw (Leica^®^SP1600, Leica Instruments GmbH, Germany). Before grinding of the sections to 30–40 µm (Struers^®^Planopol V, Merck, Germany) and surface staining with toluidine blue of the histology sections, micro-radiographs were taken (Faxitron X-ray System, Hewlett Packard, Kodak X-OMAT MA Film, Kodak, Le Plessis Grammoire, France) to visualize the stage of calcification and resorption. A high resolution analogue film was used (Kodak, Carestream Health, Inc., Rochester, NY, USA).

### 3.6. Analysis of Removal Torque-Tests

A detailed description of the removal torque testing protocol has previously been published elsewhere [56,57]. Briefly, bone blocks were embedded in dental plaster (GC Fujirock, GC Europe, Leuven, Belgium). Removal torque test was performed on a servohydraulic biaxial testing machine (MTS MiniBionix 358; MTS, Minneapolis, MN, USA). The Implant was rotated counter-clockwise at a rate of 0.1°/s to a maximum angle of 30° while simultaneously collecting angle and torque data at a sampling rate of 10 Hz. The torque-rotation curve was analyzed to define the removal torque value (N·mm) and interfacial (rotational) stiffness (N·mm/°). The complete biomechanical analysis was performed blinded.

### 3.7. Histological Analysis

**Figure 11:**
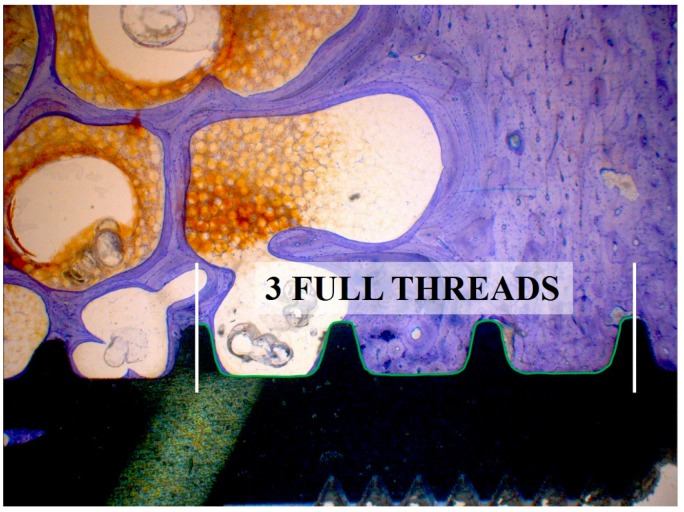
Evaluation of the bone-to-implant contact of three predefined consecutive threads on the left and right implant sides independently of existing bone quality.

**Figure 12:**
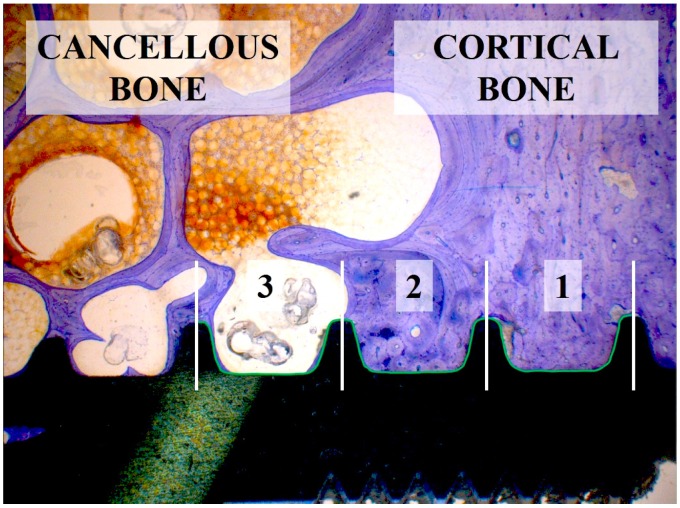
Distribution of each 3 sectors on the left and right implant side (altogether 6 sectors) to measure BIC values dependent on cancellous and cortical bone structures. Sectors 1 and 6 are coercive in cortical bone and the remaining Sectors 2–5 in cancellous bone.

Analysis of the samples was performed using a microscope (Leica^®^ M420, Leica-Microsystems, Heerbrugg, Switzerland) equipped with a digital camera (Leica^®^ DFC32). For each implant four images were captured for further analysis: overview (5× magnification), implant threads on each side (12.5× magnification) and apex (10× magnification). A semi-quantitative evaluation of the bone-to-implant contact (BIC) was conducted per implant side in longitudinal cross sections consisting of three neighboring threads (Figure 11 and Figure 12) (Right Sectors 1–3, and Left Sectors 4–6, respectively), which were subdivided into 10 sectors each. If 3 full threads were not present, no result was noted for that side.

The percentage of BIC was estimated from calibrated digital pictures in steps of 5%, independently of individual bone quality. Overall BIC values of the right and left side were summarized as “Total BIC”. In a second step the effect of cancellous (Sectors 2–5) and cortical (Sectors 1 and 6) bone structures on BIC measurements was analyzed (Figure 12). The apex was analyzed separately over the length of 4 mm (data not shown). Mean values and standard errors were calculated for Total BIC, cancellous (“Mean Cancellous”) and cortical (“Mean Cortical”) bone sectors. The complete histological analysis was performed blinded.

### 3.8. Histomorphometrical Analysis

**Figure 13:**
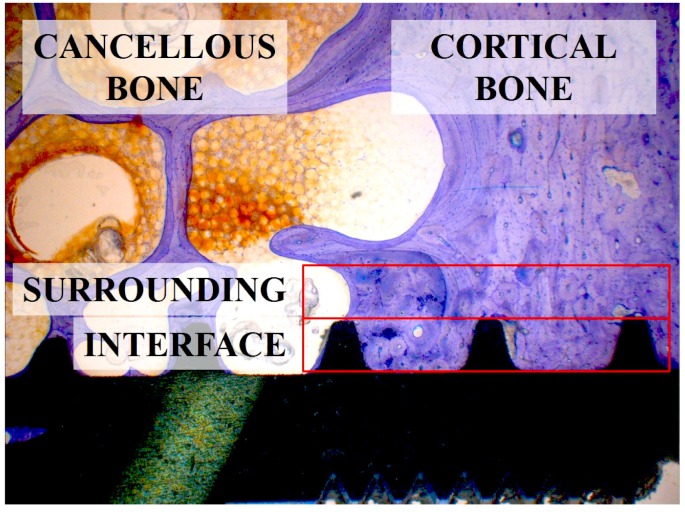
Bone compartments (interface and surrounding) for the histomorphometrical analysis.

Digital photographs were made of the toluidine blue-stained ground sections and the histological structures of interest were color-highlighted interactively with Adobe Photoshop (Adobe Photoshop Elements 3^®^ for MacIntosh). Implants were highlighted in beige, newly formed bone in green, old bone matrix in blue and bone marrow space in pink. Leica^®^QWin (Leica-Microsystems, Heerbrugg, Switzerland) was used to calculate the percentage of each tissue. In each specimen two predefined and standardized areas (3.1 mm × 0.4 mm = three full implant threads) on each implant side were evaluated separately. The first area is denominated “Interface” and covers the area within the three threads, while the second area is covering the area immediately outside of the threads and is denominated “Surrounding” (Figure 13). New bone, old bone, and bone marrow space were calculated as a percentage of the total imaged area (the area occupied by the implant was excluded from the calculation). The complete histomorphometrical analysis was performed blinded.

### 3.9. Scanning Electron Microscopy

SEM was performed on selected implants (*n =* 18) retrieved after 52 weeks healing, which have been previously used for removal torque testing. Bone blocks were stored in 70% ethanol for up to 14 months. Without further fixation, the bone block was aligned and cut along the central axis of the implant using a wheel diamond saw (thickness 300 µm). One half was removed from the bone site, critically dried under vacuum, mounted on a SEM stub and finally sputter coated with 25 nm Au/Pd layer. Prepared implants were kept under vacuum until analysis by SEM (Oxford Instruments INCASynergy 350 for combined Energy Dispersive X-ray -EDX- and Electron Back Scattered Difraction -EBSD- microanalysis incorporating INCAx-act silicon drift detector and high sensitivity/resolution NordlysS II EBSD detector). A systematic imaging of the implant surface was done at several points at the following magnifications: 15×, 65×, 100×, 250×, 1000×, 2500× (or 3500×), 5000×, 8000×, and at 15,000×, or 25,000× for special features. Some observed features appearing on the implant surface were further analyzed using EDX to determine the elemental composition. These results will be published elsewhere.

### 3.10. Statistical Analysis

Statistical analysis was carried out with the commercial software SPSS (SPSS^®^ Base für Mac OS X, Version 17.0, Chicago, IL, USA). A one-way factorial and repeated analysis of variance (ANOVA-) test was used to assess overall differences between the histomorphological parameters (Total BIC, “Mean Cancellous”, “Mean Cortical”) and the biomechanical parameters (removal torque and interfacial stiffness). Post-hoc tests according to Bonferroni served to evaluate differences between individual groups with a significance level set at *p <* 0.05.

To further explore differences between groups and to remove any potential bias introduced by the individual animal, outcome parameters were evaluated by pairing the multi-phosphonate treated implants with the corresponding control in the same animal (Real Statistics Plugin for MS Excel 2013). The data was represented in the form of a bar plot, with outcome parameter values for each multi-phosphonate treated implant normalized to the control. Individual differences between the multi-phosphonate treated implants and controls were evaluated with a one-sample two-sided Student *t*-test, with *p <* 0.05 for significance.

## 4. Conclusions 

The novel multi-phosphonate surface treatment technology was tested on commercially available titanium dental implants in an unloaded animal model. No adverse events were observed at any time point. The histological and biomechanical tests suggested that the multi-phosphonate surface treatment technology initiated bone formation and remodeling processes around the dental implants after 2, 8, and 52 weeks. The surface treatment was shown to be osteoconductive. Observation of the bone to implant interface at the cellular level by SEM analysis supported this tendency and suggested the formation of a strong bond between the multi-phosphonate treated surface and the surrounding bone.
